# History of Renal Stone Surgery: A Narrative Review

**DOI:** 10.7759/cureus.74530

**Published:** 2024-11-26

**Authors:** Taran Dhillon

**Affiliations:** 1 Medicine and Surgery, Foresterhill Health Campus, Aberdeen, GBR; 2 School of Medicine, University of Aberdeen, Aberdeen, GBR

**Keywords:** historical review, minimally invasive approach, narrative review, renal calculi (kidney stones), urological surgery

## Abstract

Untreated obstruction of the urinary tract can result in urinary stasis, hydronephrosis, and infection, which in turn lead to tissue damage, chronic renal failure, and potentially death. Renal stones have afflicted humanity throughout history, with surgical approaches evolving significantly over time. This review explores the origins and major developments in surgical techniques for renal stones, enhancing our understanding of how modern procedures have evolved. These techniques were refined over time, driven by improved anatomical knowledge and surgical experimentation. The ancient perineal lithotomy remained a standard treatment until the late 19th century, when advancements in anesthesia and antisepsis emerged. These innovations allowed surgeons to attempt more ambitious procedures, aided by early methods for visualizing internal organs. In the 20th century, innovations in radiology, imaging technologies, and surgical instruments ushered in the era of minimally invasive surgery.

## Introduction and background

Approximately 12% of the population will be affected by renal stones, with surgical approaches being recommended in cases where there is high-grade obstruction or associated infection [1]. The urinary tract has two main parts, which are the upper and lower urinary tracts. The upper tract consists of the kidney and ureters, while the bladder and urethra make up the lower tract. Stone formation can occur anywhere within the urinary tract and is the result of a chemical imbalance in the urine [2]. The process is likely to happen when there is supersaturation of the urine with crystal-forming substances such as calcium, phosphorus, and urate [3]. The risk is also increased when inhibiting factors, such as citrate, are low. Precipitated crystals can then aggregate to form a stone, which may grow large enough to cause obstruction.

Untreated obstruction of the urinary tract results in urinary stasis, hydronephrosis, and infection [4]. This can cause tissue damage, followed by chronic renal failure and death if left untreated. Depending on the location of the stone in the renal tract, pain can occur in different areas. Blockage at the site of the renal pelvis and ureter causes excruciating pain in the flank region, which can radiate to the groin [5]. This is known as renal colic and is caused by the smooth muscle of the ureter contracting tightly around the calculus. In contrast, bladder obstruction is associated with severe pain in the lower abdomen [6]. The extreme suffering caused by renal obstruction has been known by humans throughout history; hence, discovering the most effective method of removal has always been a clinical priority.

The aim of this review is to explore the origins and major developments in surgical techniques for renal stones, enhancing our understanding of how modern procedures have evolved.

## Review

Methods

Ovid MEDLINE (1946 to September 2024) and Ovid Embase (1974 to September 2024) were searched to identify potentially eligible articles pertaining to the history of renal stone surgery. The following search teams were combined: “Stone*”, “Surg*”, “Renal OR Kidney OR Ureter OR Bladder OR Urethra”, “Histor* OR Time OR Event”, “Technique OR Procedure” (*indicates truncated term). Limits added included the following: “English language”, “Full text”, “Humans”, and “Review articles”.

Following the removal of duplicates, papers, titles, and abstracts were screened and were deemed eligible if they explored surgical techniques relating to the treatment of renal stones anywhere in the renal tract throughout time. If full-text articles were not available or abstracts were not available in the English language, they were not cited. Only review articles were searched, and studies pertaining to animals but not humans were excluded. Additional articles and sources, including books and online publications, were found in the reference list of review articles.

After the application of limits and deduplication, a total of 687 unique review articles were found. Of these, 33 review articles were chosen. A further 22 online journal publications, seven books, and three websites were cited.

Perineal lithotomy

**Figure 1:**
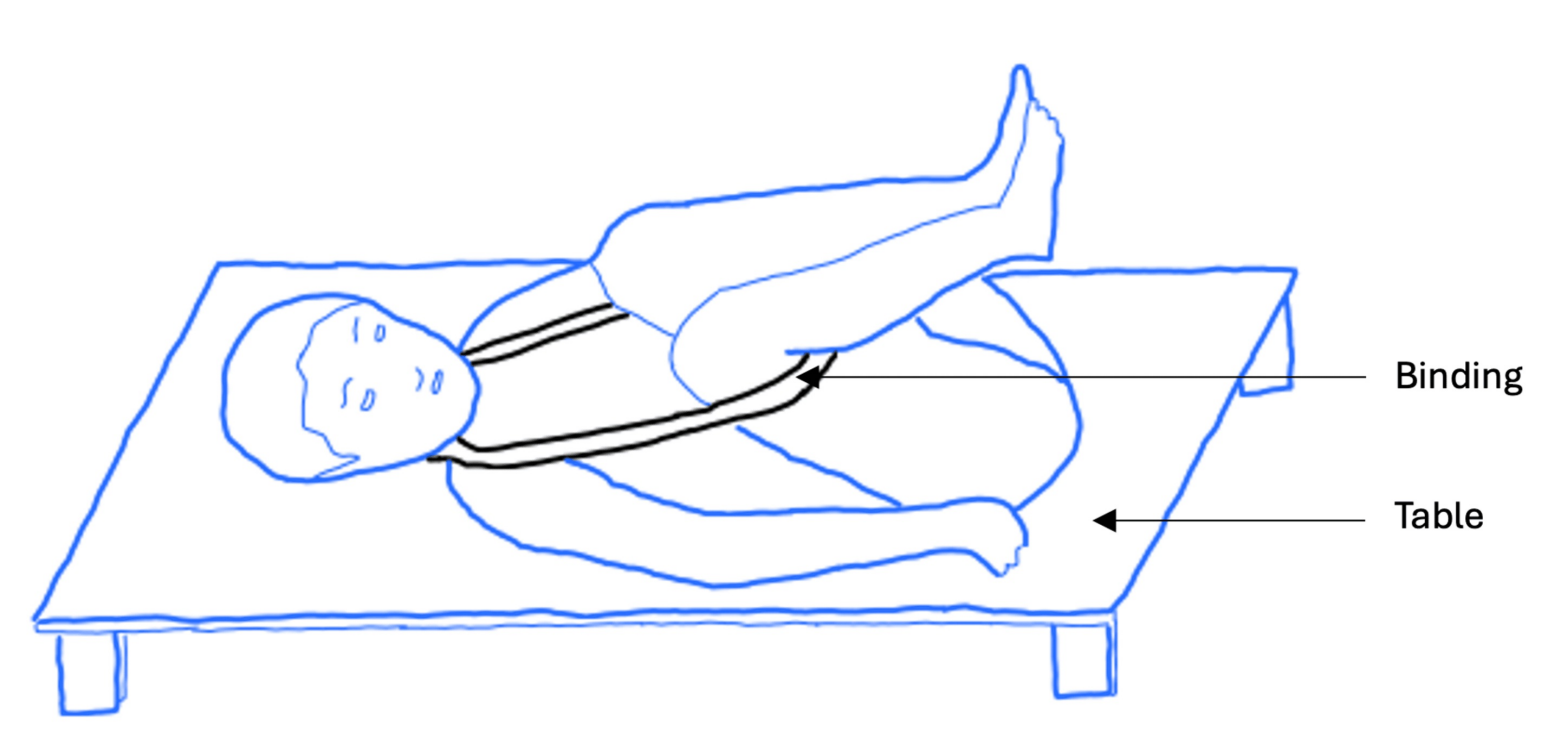
Perineal lithotomy as described by Sushruta The patient was positioned in the lithotomy position, with their legs bound by cloth. The physician then attempted to pull the stone inferiorly, to the bladder neck, for easier access. This was done by applying strong pressure to the hypogastric region while simultaneously passing the index and middle fingers into the rectum and pulling down on the stone. In women, two fingers were inserted into the vagina instead, due to its closer relation to the bladder. An incision was then made adjacent to the perineal raphe, the length of which corresponded to the stone size. The bladder was incised, and the stone was removed using a curved spoon. The wound was cauterized in the event the micturition did not return to normal after a week [9]. Image created using Sketch.IO

The earliest description of a surgical approach for removing bladder stones dates back to 600 BC [7]. Sushruta, a surgeon living in India, provided detailed instructions for his method. He was ahead of his time and placed great importance on anatomical knowledge when performing surgery. He acknowledged the ureters, bladder, and urethra and their role in conveying urine to the outside environment [8]. Knowing this, he recognized urinary stasis as a key symptom of stone obstruction, alongside severe abdominal pain. His procedure was described in detail (Figure 1).

Sushruta recognized the high mortality of surgery, so he always favored less invasive approaches and only operated in the event other treatments failed [10]. This included herbal remedies, diuretics, and bladder irrigation with a transurethral device. Despite this, many patients eventually progressed to chronic obstruction, and the pain of surgery seemed worthwhile in the hope of relief. Rhazes of ancient Iran (865-925 AD) proposed several new advancements in treating renal stones. He performed lithotomies similar to Western practice at the time and also, for the first time, described the use of enemas to reduce the risk of infection before surgery [11]. He emphasized the prevention of stones, advising to avoid rich foods. In terms of pain management, he prescribed sedatives to ease the pain of ureteric colic, which he recognized was due to stones passing through the ureters. To relieve the pain of urinary retention, he utilized a catheter with eyelets on the side as opposed to the end. These developments are still found worldwide today, and his influence on modern practice is clear.

Early medical management of urinary stones was limited, most likely secondary to poor understanding of the underlying causes of stones. Common treatments included diuretics (to increase urine flow) and tonics thought to “dissolve” stones or relieve pain, although with limited effectiveness [10]. However, in Ancient China, herbal medicines such as “Wu-Ling-San” have been used to prevent stones since the first century BC. They are plant-based compounds primarily composed of carboxyl, hydroxyl, amino acid groups, and oxygen-containing heterocycles, which can form coordination complexes with calcium ions [12]. This, in turn, helps reduce the supersaturation of urine with calcium ions, a known contributor to the formation of urinary stones. A cohort study demonstrated the efficacy of "Danshen,” a traditional Chinese medicine, in preventing urolithiasis [13]. These compounds continue to be studied today for their potential in reducing kidney stone recurrence and aiding in treatment post-surgery, as their mechanism of action is still not fully understood.

The Western practice of perineal lithotomy introduced several variations on the procedure. Celsus of Rome (25 BC-AD 50) described the start of the procedure similarly to Sushruta, although his descriptions of perineal incisions were more detailed [14,15]. The first incision was made in the shape of a crescent with the ends angled toward the ischial tuberosities. The depth of the cut extended to the neck of the bladder. A second transverse incision was then made into the bladder neck, and the stone was retrieved using a hook. This technique was known as “operation minor” due to the limited number of surgical instruments required to perform it [16]. Later developments by Romano in 1520 brought several new instruments to aid in accessing the bladder neck (see [17], p. 188 for a full list of instruments). Romano passed a grooved staff into the urethra, which allowed other instruments to be guided along its groove. Instead of a scalpel, Pare’s dilator was used to open the prostate and bladder neck, followed by forceps to retrieve the stone. Common complications included major hemorrhage, fistulas, and incontinence [18]. Despite this, perineal lithotomy continued to be practiced throughout the Renaissance period.

Frere Jacques (1651-1714), a traveling lithotomist, used a lateral incision to access the bladder, as he described it as being easier to reach the stone [19]. His mortality rate was high, at 53.5% from 71 consecutive operations [19]. He has been described as a “rogue lithotomist” owing to his lack of anatomical knowledge and poor post-op care [17]. On one day, seven of his patients died, and so he accused the monks in the monastery of poisoning his patients. However, an autopsy revealed grave wounds to the bladder and rectum of his victims [17]. His unethical practice was further highlighted by the fact that he would allow large crowds of onlookers to purchase tickets to observe his practice. There was a lack of standardization of practice secondary to surgery being viewed as inferior to the medical field at the time. This was in part due to the Hippocratic Oath stating the following [20]: “I will not cut for the stone, even for patients in whom the disease is manifest; I will leave this operation to be performed by practitioners.”

Furthermore, surgery had just become recognized as an academic discipline during the Renaissance period. During medieval times, there was an abundance of superstitious beliefs that discouraged physicians from performing surgeries [21]. Hence, the role of the surgeon was delegated to “barbers” and was not highly regarded in intellectual medicine. As surgical knowledge became more respected and scientific approaches were encouraged, surgeons gained more formal education and gradually became recognized as medical professionals. This may be due in part to improved anatomical knowledge secondary to pioneers like Andreas Vesalius publishing detailed anatomical studies, challenging and correcting Galen’s errors, which were heavily relied upon during the medieval period [22]. The advent of the printing press also allowed a wider distribution of medical texts and illustrations so surgeons could learn from others’ discoveries.

With the advent of general anesthesia during the 19th century, great suffering was alleviated. Now looking back, surgery in the past included many practices that would be unacceptable today. Advances in medical knowledge and ethical understanding have led to major improvements in patient care and protection, transforming surgery into a safer and more respectful practice. Perineal lithotomy was described as “one of the most terrible in surgery” by later lithotomists, and the procedure itself was compared to a “ferocious battle” [17]. A patient’s first-hand account of the procedure describes surviving the operation through his faith in God [20]. So what other developments would lead to improvements in surgical care?

The advent of anesthesia and antisepsis

Two major changes advanced surgical management of renal stones during the 19th century. Chloroform started to be widely used in 1847 and provided full-body anesthesia, allowing a stable surgical field, reducing patient movement, and improving surgeon control [23]. Nowadays, improvements in anesthetic drugs and better monitoring techniques have led to major improvements in mortality, with specifically anesthesia-related deaths dropping from 357 per million before the 1970s to 34 per million in the 2000s [24]. The second major change was the increased uptake of asepsis around 1867, when Lister discovered carbolic acid was effective in preventing wound gangrene. Since then, there has been a reduction in surgical site infections by up to 50-70% [25]. Sepsis accounts for approximately 50% of mortality due to urolithiasis, especially in the postoperative period; hence, addressing this complication was of great importance [26].

These changes allowed surgeons to experiment with a new variety of techniques to treat urinary stones with better efficacy. Civiale (1792-1867) was one of the earliest urologists to demonstrate the superiority of a treatment through comparison of relative mortalities. He showed that transurethral lithotripsy had a mortality rate of 2.3% (n = 257) compared to perineal lithotomy, which had a 20% mortality rate (n = 5,715) [27]. The major cultural shift toward humanism and scientific inquiry during the Renaissance period led to a more empirical approach to surgery, fostering a greater reliance on evidence and direct experience to advance surgical technique. The method of transurethral lithotripsy has its origins around 1000 AD, when Arab surgeon Albucasis described using a drill passed through a metal catheter to fragment stones within the urethra [28]. However, widespread uptake of the technique would not occur until after the publication of Civiale’s findings.

A more sophisticated calculus crushing mechanism was devised by Herteloup for the transurethral approach around 1830 [29]. Once the stone was grasped, a screw was rotated by a wheel at the proximal end of the device, thus crushing the stone in a controlled manner. The method was perfected by Bigelow, who took full advantage of general anesthesia [30]. The length of the procedure could be greatly increased, providing additional time to remove all stone fragments. Bigelow created the “Evacuator,” consisting of a catheter attached to a bulb at its proximal end, which removed stone fragments via suction (see [31,32] for images of Bigelow’s instruments).

Open approaches to renal stone surgery were also developed for the treatment of larger bladder stones. One of the earliest suprapubic lithotomies was carried out on an infant in 1560, where a large stone was removed successfully [33]. Discovery of the lower peritoneal boundaries in 1718 meant surgeons could now expand the bladder to force the peritoneum superiorly and avoid incising it during surgery [34]. Additionally, the introduction of anesthesia and antisepsis toward the end of the 19th century meant a suprapubic approach became more effective [35]. Muscle relaxants made it easier to incise the abdomen, and the bladder could be filled to a greater extent. This meant the peritoneum could be lifted higher in the abdomen and safe from incision. The use of aseptic techniques reduced infection rates, and the self-retaining balloon catheter, introduced by Reybard in 1855, minimized bladder wound complications [36]. Consequently, mortality rates of suprapubic lithotomy significantly decreased as a result.

**Figure 2:**
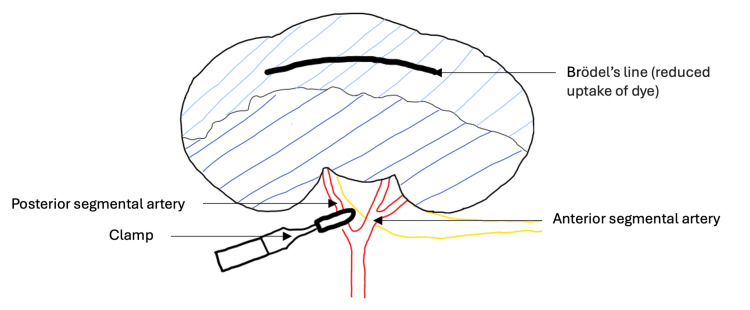
Brödel’s line/renal avascular plane during anatrophic nephrotomy An accurate position of Brödel’s line is determined by clamping the posterior segmental artery and injecting the patient with IV methylene blue. A diathermy needle is then used to outline the segmental boundary. The clamp is removed and placed over the renal artery. A shallow incision is then made into Brödel’s line, followed by blunt dissection to reach the renal collecting system. The calyces are then opened to expose the calculi [41]. Image created using Sketch.IO

Surgical techniques had been limited to the bladder and urethra before the advent of anesthesia and antisepsis. One of the earliest successful surgical approaches to kidney disease was described by Simon in 1871 [37]. This was a nephrectomy whereby an oblique incision was made from the 12th rib to the iliac crest. The exposed kidney was then cut out and open blood vessels ligatured. Variations in the procedure included nephrolithotomy described by Morris in 1880 and partial nephrectomy described by Bardenheuer in 1891 [38,39]. The next significant development in the surgical management of calculi within the kidney occurred in 1901, when Brödel identified the renal avascular plane by injecting colored dye into the renal segmental arteries [40]. The plane is relatively avascular because the renal end arteries do not anastomose. Surgeons greatly benefited from this discovery, as it allowed for reduced blood loss when accessing the renal calyces. Consequently, anatrophic nephrotomy was developed. This procedure minimized parenchymal loss by creating an incision through the avascular plane (Figure 2). While anatrophic nephrotomy is still performed in most cases requiring open surgery, the demand for open surgery has significantly declined due to the rise of minimally invasive treatment options.

**Figure 3:**
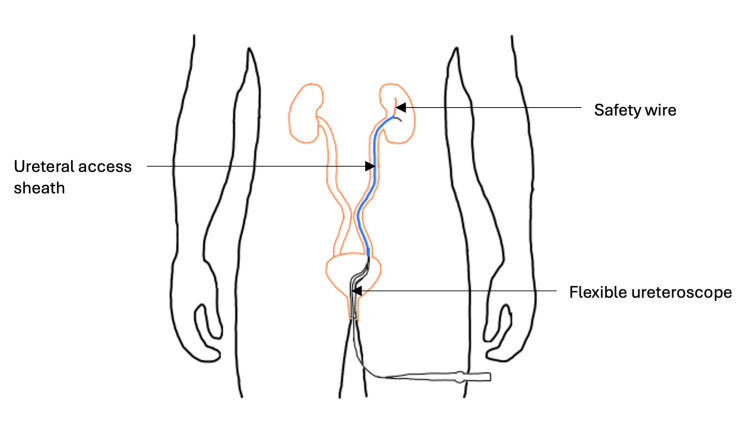
UAS enabled access to the upper renal tract UAS facilitated the safe insertion of ureteroscopes into the ureter and kidney [44]. UAS, ureteral access sheaths Image created using Sketch.IO

Visualization of stones within the renal system was the next major development in the less invasive approach to renal stone management. In 1877, the earliest cystoscopy device was created by Nitze and was later improved by the Edison lamp a decade later [42]. Young integrated the cystoscope with the lithotrite in 1908, allowing surgeons to locate and crush stones with less difficulty [43]. Developments in ureteroscopy occurred in 1974 when Takayasu et al. devised the first ureteral access sheaths to facilitate the insertion of ureteroscopes into the ureter (Figure 3).

**Figure 4:**
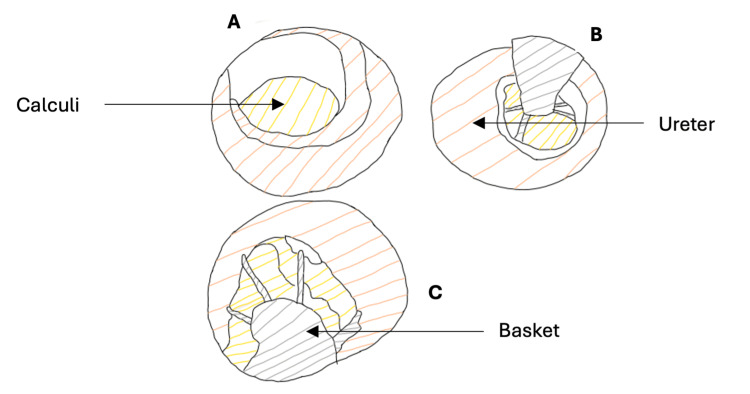
Removal of ureteric calculi via ureteroscopy (A) Visualization of the stone using the ureteroscope. (B) Retrieval of the stone using the basket system. (C) Removal of the stone from the urinary tract. If there is difficulty in removing the stone using the basket, it can be fragmented via ultrasound or laser. A stent may be left to ensure the kidneys drain well [46]. Image created using Sketch.IO

Ureteroscopes with a basket retrieval system were used effectively in patients from 1981 with ultrasonic probes added in 1983 [45] (Figure 4).

In 1989, the holmium laser was introduced, and it had several advantages over ultrasound [47]. It was powerful enough to fragment the hardest stones and could be emitted from the narrowest fibers. This made it well suited for passing through flexible ureteroscopes and could reach as far as the superior pole of the kidney. Following technological breakthroughs, global contributions have led to significant developments in the field of endourology over the last century.

**Figure 5:**
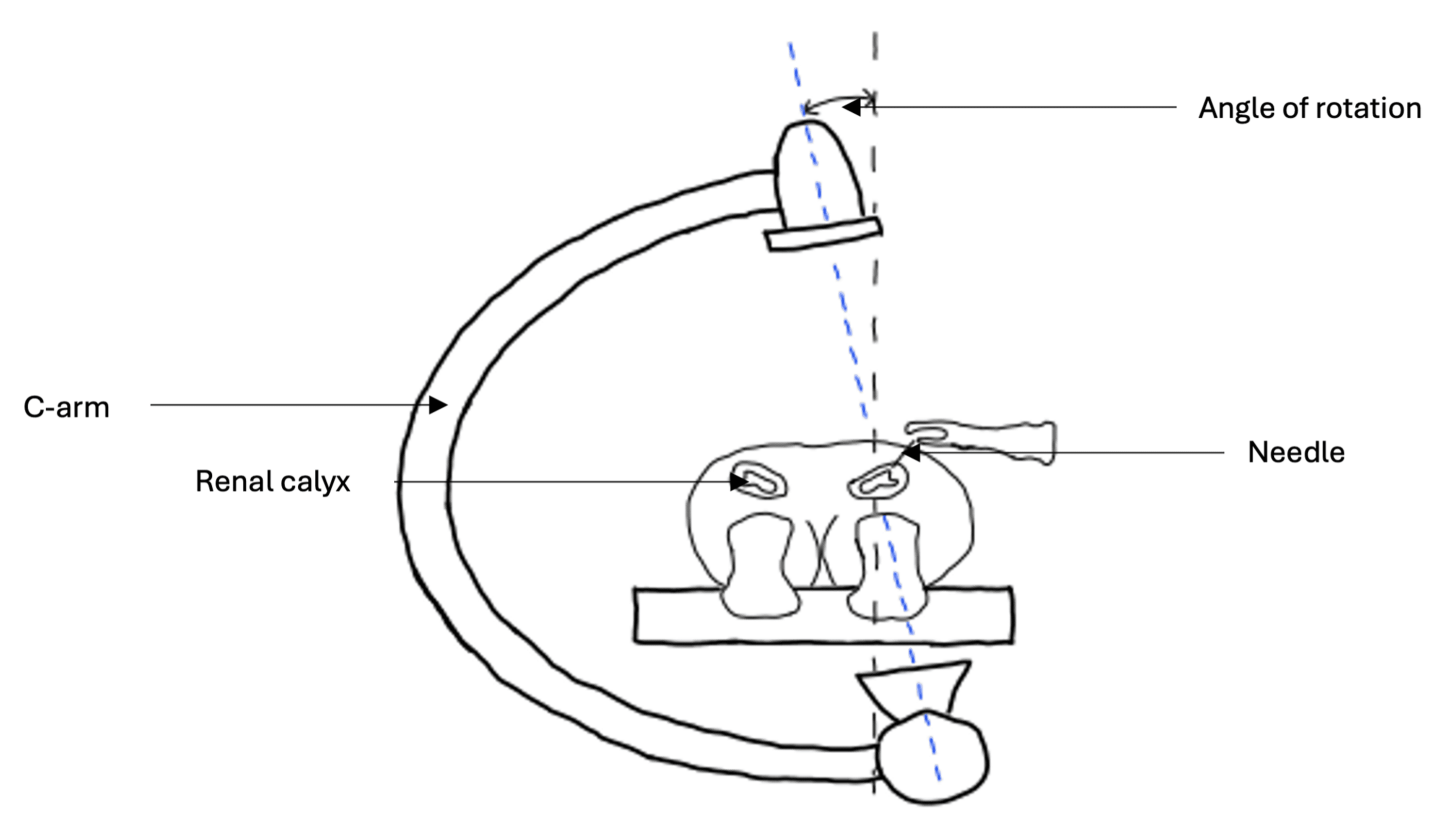
Needle puncture under fluoroscopy guidance during PCNL The patient is placed in the prone position, and fluoroscopy is used to guide needle puncture. The c-arm of the X-ray machine is rotated to ensure the correct positioning of the needle when entering the desired calyx. A guide wire is then passed through the needle into the renal calyx. The needle is removed, and a dilator of appropriate size is passed along the guide wire alongside an access sheath of similar diameter [51]. PCNL, percutaneous nephrolithotomy Image created using Sketch.IO

**Figure 6:**
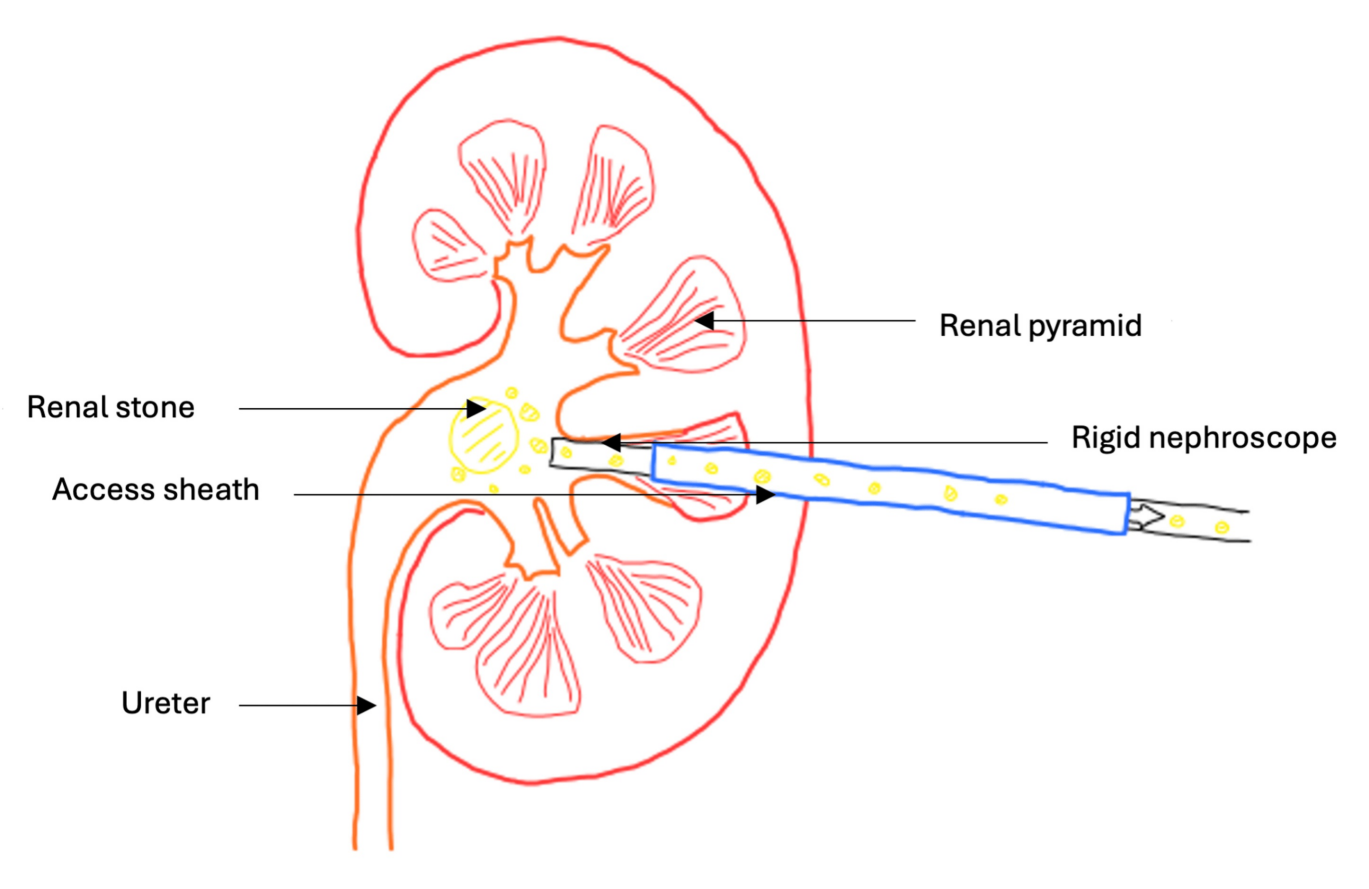
Renal stone removal during PCNL A nephroscope is inserted, and stones are fragmented. A ureteral catheter helps remove any stone fragments through high-pressure irrigation of the collecting system. Through modification of nephrostomy tract diameter, a wide variety of stone sizes can be treated [52]. PCNL, percutaneous nephrolithotomy Image created using Sketch.IO

Another major advancement in surgical management was the introduction of percutaneous nephrolithotomy (PCNL). This method has its origins when Goodwin established the first nephrostomy tract in 1955 when inserting a tube into the renal pelvis of a hydronephrotic kidney [48]. Following this, many started to consider the possibility of tract dilation to aid stone removal. With fluoroscopy guidance, the first percutaneous extraction of renal calculi was performed in 1976 [49]. Dilation of the tract was achieved with polythene dilators, and the stone was extracted using a basket system. The popularity of PCNL soared during the 1980s when Amplatz and Smith designed a balloon dilator able to pass smoothly along a guide wire, thus shortening the dilation time [46]. A nephroscope integrated with an ultrasonic probe proved effective in treating staghorn calculi, further contributing to increased uptake of PCNL [50] (Figure 5, Figure 6).

Developments on standard PCNL include minimally invasive PCNL, which utilizes smaller tracts and sheaths to aid in patient recovery. Zeng et al. discovered that super-mini PCNL is a safe and effective treatment for renal stones of <2.5 cm and is suitable for patients with lower pole stones and stones not amenable to retrograde intrarenal surgery [53]. Furthermore, the needle perc technique, which utilizes narrower fibers, could be used for smaller stones; however, complex stones still require conventional tracts [54]. Meta-analysis data has shown that stone-free rates with minimally invasive PCNL can reach up to 84%, similar to standard PCNL [55]. Additionally, minimally invasive PCNL demonstrated reduced hospital stay and fewer complications compared to standard PCNL. More recent developments include the technique of endoscopic combined intrarenal surgery. This combines PCNL with retrograde intrarenal surgery to improve precision and safety in complex cases. Hamamoto et al. found that stone-free outcomes reached up to 70% in patients with staghorn calculi [56]. The current aim of renal stone surgery is to minimize invasiveness, but what treatments are used to reduce the need for surgical intervention?

Noninvasive approaches to renal stone management

During the 1970s, a revolutionary discovery was made that would dramatically change renal stone management. It was found that shockwaves caused no obvious injury when passing through muscle or fat, so naturally, urologists grew curious about the efficacy of shockwaves in stone fragmentation [57]. The first patient was treated successfully with the Dornier HM-1 lithotripter in 1980, and since then, millions of patients worldwide have received the same treatment [57]. Extracorporeal shockwave lithotripsy (ESWL) was game-changing in the treatment of renal calculi, as before its discovery, patients with only a minor stone burden would have undergone invasive treatment. This method is most effective in treating renal and ureteral calculi less than 2 cm in diameter [58]. Another major advantage of ESWL is that general anesthesia is not required. Before ESWL, the only noninvasive treatment options available were medical therapies.

The development of more effective medical therapies for renal stones coincided with increased knowledge of stone formation. During the Renaissance period, there was a renewed interest in mineral chemistry, which contributed to a better understanding of urinary stones [59]. Additionally, physicians recognized the link between diet and stone formation. Recommendations to avoid specific foods, such as those high in purines, e.g., red meat, became more common. Later in the 20th century, alkaline compounds, such as potassium citrate, were prescribed to make urine less acidic, which could prevent certain types of stones from forming [1]. Specific types of stones were also identified, including calcium oxalate, uric acid, and cystine stones. It was recognized that each stone type required a different approach to treatment. During the 20th century, researchers developed medications to dissolve specific types of stones. For uric acid stones, medications like allopurinol were introduced to reduce uric acid levels [60]. For recurrent calcium stones, thiazide diuretics became popular as they helped lower calcium levels in urine. These targeted therapies represented a breakthrough in both the prevention and management of stones.

Medical treatments have reduced the need for invasive intervention in the modern day, with only 10-15% of renal stone cases needing surgical intervention nowadays [61]. In the late 20th century, treatment focus shifted to understanding metabolic and genetic causes of stone formation, leading to more personalized approaches to prevention. For example, genetic studies furthered the understanding of rare stone types, like cystinuria, leading to even more personalized treatments [62]. Modern treatments often involve a combination of lifestyle, dietary adjustments, and medications tailored to the patient’s specific stone type and risk factors. Medications like thiazides, potassium citrate, and allopurinol remain central to prevention and have been shown to reduce recurrence rates, size, and severity of stones [60]. When surgery is needed, medical management is often used in conjunction to break down stones into smaller fragments before surgery. Meta-analysis data has shown alpha-blockers to be an effective first-line therapy for the treatment of stones less than 10 mm in diameter [63]. For larger stones or where there is significant hydronephrosis or infection, active surgical intervention is then recommended.

Future directions

The future of renal stone surgery is continually growing with technological innovation. Artificial intelligence (AI) could assist in diagnostics, planning, and the surgical process itself, leading to improved patient outcomes. Through improved radiological image interpretation and the formation of detailed 3D models, AI can assist in determining the exact position, size, and shape of the stones or other structures, aiding surgical planning and reducing complications [64]. It could also aid in risk assessment by analyzing a patient’s comorbidities and past imaging results to assess the risks of different surgical options [65]. During the surgery itself, robotic machines such as the “da Vinci Surgical System” could be enhanced with AI to allow finer control and more efficient removal of stones, minimizing tissue damage as a result [66]. Furthermore, robotics has been expanding the possibilities of minimally invasive approaches through robotic-assisted ureteroscopy and PCNL, leading to improved clinical outcomes [67].

In the future, biotechnological approaches such as gene therapy may offer future solutions for managing genetic predispositions to stones. By targeting specific mutations in genes at the cellular level, such as those affecting calcium and oxalate metabolism, reduction of these elements could decrease the likelihood of stone formation. Advances in our understanding of the monogenetic basis of cystinuria make it an attractive model of study; however, nanoparticle and viral vector gene delivery remains a challenge due to complex renal tissue architecture [68]. More promising is the use of precision medicine, which uses genetic screening to identify patients with a higher genetic predisposition to certain types of kidney stones. For example, in cystinuria, a vegan diet and potassium citrate supplementation are recommended to achieve urinary alkalinity [69]. Whereas in adenine phosphoribosyl transferase deficiency, allopurinol is recommended. This personalized approach to stone prevention could be further enhanced by AI to predict the likelihood of stone recurrence and recommend specific dietary changes, medications, or lifestyle adjustments to minimize risk on an individualized basis [70]. Another cutting-edge avenue is the use of nanotechnologies in stone fragmentation through the use of elemental nanoparticles, which has only been tested in concept [71].

## Conclusions

This narrative review traces the evolution of renal stone management throughout history. Surgical techniques for removing urinary stones date back as far as 600 BC. Perineal lithotomy, although widely used, was a feared procedure due to its high risk and lack of anesthesia or antisepsis. The advent of anesthesia and antiseptic techniques in the 19th century revolutionized stone surgery, enabling safer and more effective methods, such as endourological approaches, PCNL, and anatrophic nephrolithotomy. The 20th century saw rapid advancements driven by improvements in radiological imaging and surgical equipment. The introduction of ESWL marked a turning point in noninvasive management. By examining these historical milestones, it is evident that better control over surgical outcomes encouraged the development of more ambitious and sophisticated techniques. The cultural shift from superstition to scientific inquiry also played a crucial role in driving innovation and education in the field.

Medical management has long been a first-line approach. Ancient treatments included herbal medicines and diuretics, while modern advancements have introduced evidence-based therapies and personalized approaches through genetic testing. In the future, AI technology is expected to play a growing role in both the medical and surgical management of renal stones. Gene therapies have shown promise, though further research is needed to optimize delivery methods. Similarly, the potential of nanotechnology in stone fragmentation awaits real-world testing. Today, only a minority of patients require invasive treatments. Unlike in ancient times, when options were limited and fraught with danger, modern techniques can effectively treat all types of stones in any part of the renal tract.
